# Comparative meta-analysis of task-related functional brain abnormalities in nonsuicidal self-injury and suicide attempt

**DOI:** 10.3389/fpsyt.2026.1803658

**Published:** 2026-07-07

**Authors:** Lu Tang, Xiqin Liu, Yuanyuan Li, Nanfang Pan, Jianyu Li, Jian Zhou, Benjamin Becker, Qiyong Gong

**Affiliations:** 1 Department of Radiology, Huaxi Magnetic Resonance (MR) Research Center (HMRRC), Functional and Molecular Imaging Key Laboratory of Sichuan Province, West China Hospital of Sichuan University, Chengdu, Sichuan, China; 2 Research Unit of Psychoradiology, Chinese Academy of Medical Sciences, Chengdu, Sichuan, China; 3 Xiamen Key Laboratory of Psychoradiology and Neuromodulation, Department of Radiology, West China Xiamen Hospital of Sichuan University, Xiamen, Fujian, China; 4 Department of Radiology, Affiliated Hospital of Guizhou Medical University, Guiyang, Guizhou, China; 5 State Key Laboratory of Brain and Cognitive Sciences, The University of Hong Kong, Hong Kong, Hong Kong SAR, China; 6 Department of Psychology, The University of Hong Kong, Hong Kong, Hong Kong SAR, China

**Keywords:** functional magnetic resonance imaging, nonsuicidal self-injury, psychoradiology, seed-based d mapping with permutation of subject images, suicide

## Abstract

**Background:**

Nonsuicidal self-injury (NSSI) and suicide attempt (SA) are two major self-injurious behaviors causing substantial suffering and socioeconomic burden. However, it remains unclear whether NSSI and SA are characterized by common or distinct brain dysregulations. Here, we aimed to identify shared and separable neurofunctional alterations during task engagement between NSSI and SA.

**Method:**

A coordinate-based meta-analysis was employed using Seed-based d Mapping with Permutation of Subject Images (SDM-PSI) by capitalizing on task-based functional magnetic resonance imaging (fMRI) studies comparing the brain activation between NSSI/SA individuals and controls.

**Result:**

The search identified 10 studies for NSSI (n = 200, mean age: 22.89 years) and 16 studies for SA (n = 343, mean age: 28.65 years). After threshold-free cluster enhancement correction, NSSI individuals exhibited increased right amygdala activation relative to both controls and the SA group, as well as heightened left middle frontal gyrus and reduced left paracentral lobule activation compared to the SA group. No significant activation differences were found between SA and controls, though a less conservative threshold revealed increased left postcentral gyrus activation in the SA group. No shared functional abnormalities were identified between NSSI and SA under either corrected or uncorrected thresholds. Importantly, subgroup analyses revealed that the neurofunctional abnormalities in NSSI were primarily driven by adolescent cohorts, whereas no significant clusters emerged for the SA group across age-stratified analyses.

**Conclusion:**

These results suggest that neurofunctional abnormalities are evident in adolescent NSSI, particularly in fronto−limbic regions, with no robust findings in adult NSSI or SA subgroups. This highlights a unique developmental trajectory that necessitates age-tailored risk assessment and interventions for self-injurious behaviors.

## 1 Introduction

Non-suicidal self-injury (NSSI) and suicide attempt (SA) are two major public health issues worldwide, resulting in significant suffering and socio-economic costs. Both behaviors represent self-injurious behaviors (SIB), reflecting direct and intentional harm to oneself, and are closely related to psychiatric conditions (1–3). Although NSSI and SA are both forms of SIB, they differ in several aspects including intention, repetition, and lethality (4). NSSI is characterized by the repetitive engagement in deliberate self-harm behaviors, including the destruction or manipulation of bodily tissues without any suicidal intention (5), which generally serves as an emotion regulation strategy to alleviate unwanted emotional arousal or reduce psychological distress (6). SA is a self-destructive and impulsive behavior aimed at ending one’s own life (7), often motivated by profound hopelessness or perceived burdensomeness (8). It has been reported that NSSI and SA often co-occur (9, 10), and a history of NSSI is one of the strongest predictors of SA based on cross-sectional (11) and longitudinal (12) evidence. Overlapping genetic factors have also been found in NSSI and suicidal self-injury, suggesting that they share biological underpinnings (13). Despite the fact, the fifth edition of the Diagnostic and Statistical Manual of Mental Disorders (DSM-5) has established separate diagnostic entity for NSSI (14, 15). Although there are still ongoing discussions regarding the etiological and nosological distinctiveness of NSSI and SA (15), their unique and shared neurobiological underpinnings remain poorly understood.

Human neuroimaging studies have provided preliminary yet heterogeneous findings on the neural correlates of NSSI and suicidality. Initial evidence suggests a close relationship between NSSI and limbic hyperarousal and prefrontal regulation dysfunction, including overactivity of the amygdala, insula, dorsolateral prefrontal cortex (dlPFC) and anterior cingulate cortex (ACC) (16, 17), and SA has been shown increased activation in the ACC, superior frontal gyrus, and middle frontal gyrus (MFG) (18, 19). Recent meta-analysis of functional magnetic resonance imaging (fMRI) studies demonstrated heightened activation in the right medial frontal gyrus and left inferior frontal gyrus (IFG) in NSSI individuals compared with healthy controls, which may be related to their deficits in emotion regulation and reward processing (20). In contrast, whole-brain meta-analysis of individuals with suicidal ideation or suicide attempts showed increased activation in the bilateral superior temporal gyrus, left middle temporal gyrus, and bilateral middle occipital gyrus, and decreased activation in the right putamen and left insula compared with nonsuicidal controls (21). Dysfunctions in these regions may be associated with high impulsivity and disturbed emotional and cognitive processing in suicidal individuals (21). A recent review by Auerbach et al. (22) elucidated that both suicidal thoughts and behaviors (STB) and NSSI are related to top-down and bottom-up neural alterations such as reduced ACC volume and blunted striatal activation, while STB is particularly characterized by changes in prefrontal regions such as decreased volume in ventral prefrontal cortex and orbitofrontal cortex, and NSSI is more associated with amygdala disturbances which contributes to hyperarousal (22). These findings suggest that there may be shared and independent brain changes between NSSI and suicidality. However, due to the lack of research directly comparing NSSI and suicidal individuals, it is currently unclear to what extent specific or common brain activation alterations occur in one group versus the other. Addressing this issue may help to elucidate the neural differentiation between NSSI and suicidal behaviors and develop more targeted brain markers, thereby improving early identification and treatment strategies.

Against this background, the present coordinate-based meta-analysis aimed to compare the brain activations in NSSI and SA using previously conducted task-related fMRI case-control studies via Seed-based d Mapping with Permutation of Subject Images (SDM-PSI) version 6.21 (https://www.sdmproject.com/), a novel and well-established method of performing neuroimaging meta-analyses that allows highly robust statistical inference by threshold-free cluster enhancement (23). Importantly, the present study focuses specifically on SA rather than suicidal ideation, because SA, like NSSI, is an overt self-harm behavior, whereas suicidal ideation remains at the cognitive level and does not necessarily involve action (24). Directly comparing two behavioral phenotypes (NSSI and SA) allows for a more interpretable contrast of the neural mechanisms underlying actual self-harm behaviors. To determine common and distinct neurofunctional alterations between NSSI and SA individuals, we (1) conducted separate meta-analyses within each group to characterize robust activation abnormalities relative to healthy controls; (2) performed quantitative comparison of activation abnormalities between NSSI and SA; (3) assessed overlapping regions of shared abnormalities across the two groups; and (4) examined the potential confounding effects of demographic and clinical conditions via several complementary analyses.

## 2 Methods

### 2.1 Search and study selection

**Figure 1:**
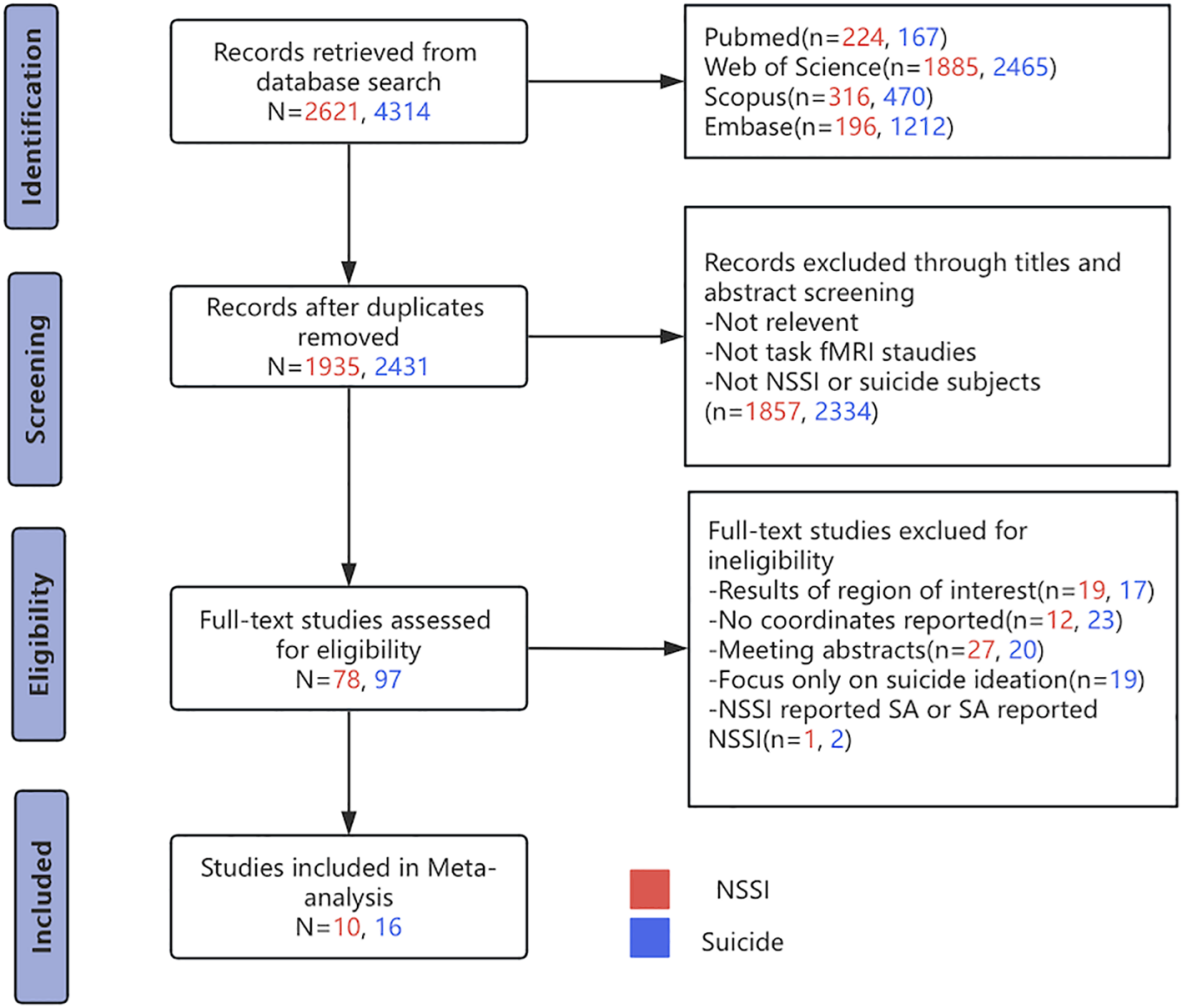
PRISMA flow diagram of study selection in the current meta-analysis. NSSI, nonsuicidal self-injury; SA, suicidal attempt.

A systematic literature search was conducted in PubMed, Web of Science, Scopus and Embase databases for case-control whole-brain studies of NSSI and SA using task-fMRI published up to May 2025 (**Figure 1**), according to the PRISMA guidelines (25). The key search terms were: ‘functional magnetic resonance imaging OR fMRI’ in combination with either ‘nonsuicidal self injury’ OR ‘suicide’. Note that broad search terms were used to prevent missing any relevant studies. Studies focusing solely on suicidal ideation without a history of SA were excluded because this meta-analysis aims to perform direct neurofunctional comparisons between actual self-harming actions rather than contemplation of self-harm. Detailed search terms are provided in **Supplementary Table 1** in **Supplementary Material**.

Studies meeting the following criteria were included: (1) reported brain activation differences between individuals with a history of nonsuicidal self-injury or suicide attempts and controls (patient controls, PCs or healthy controls, HCs); (2) providing coordinates in Talairach or Montreal Neurological Institute (MNI) space; (3) performing whole-brain analyses; and (4) fMRI studies published in English. Studies were excluded if (1) only a region-of-interest approach was used; (2) task-fMRI was not used to compare brain activation between NSSI/SA and controls; (3) peak coordinates were not reported; (4) NSSI was explained by other diseases, such as skin picking disorder or encephalitis; (5) SA individuals had a history of NSSI, or NSSI individuals had a history of SA within the past 12 months. Missing data (e.g., sample size etc.) was requested from authors via e-mail.

Data were independently screened and extracted from all eligible studies by two reviewers (X.Q.L and L.T) until 100% agreement was achieved. For each study included in this meta-analysis, we recorded the peak coordinates and effect sizes of clusters with significant activation in both directions (i.e., NSSI/SA > controls, and NSSI/SA < controls) as well as other basic information (e.g., sample size, psychiatric condition, age, sex, threshold etc.).

### 2.2 Meta-analysis

All analyses were implemented in SDM-PSI version 6.21 (https://www.sdmproject.com/). Using original peak coordinates or whole-brain *t-*maps, SDM-PSI recreated unbiased effect size maps based on Maximum likelihood estimation and the MetaNSUE algorithm (26, 27). All maps were combined with a standard random-effects modeling, accounting for sample size, intra-study variability and between-study heterogeneity. Furthermore, family-wise error (FWE) correction with threshold-free cluster enhancement (TFCE) was used to improve the reliability of the meta-analytic results.

To investigate common and distinct neurofunctional alterations between NSSI and SA, we used a three-step meta-analytic approach: (1) first, we performed separate meta-analysis within each group (i.e., NSSI vs controls, SA vs controls) to examine robust activation or deactivation for each group in comparison to all controls; (2) to characterize distinct functional abnormalities, we conducted a comparative meta-analysis between NSSI and SA (NSSI vs SA, relative to their respective controls) covarying for age and sex; (3) a conjunction analysis was performed to assess overlapping activation abnormalities across NSSI and SA.

To explore the effects of potential confounding factors, meta-regression analyses were conducted to examine whether the regions identified from separate meta-analyses were associated with variables that met the minimum requirement of ≥ 9 studies for inclusion in the meta-regression (28). Subgroup meta-analyses based on age (pediatric or adult), control type (PCs or HCs), psychiatric condition (e.g., major depressive disorder, MDD) and task type (emotional or cognitive tasks) were performed to validate the stability of the results and address the heterogeneity of the included studies. All meta-analyses were thresholded at TFCE-based FWE corrected *p* < 0.05 with a voxel extent ≥10 to control for multiple comparisons and obtain robust findings. In line with previous studies (29–31), a classic threshold (uncorrected *p* < 0.005 and cluster extent ≥ 10 voxels) was also employed to balance sensitivity and specificity for exploratory analyses, including the separate meta-analyses and the conjunction analysis. Notably, this exploratory threshold was not applied to the comparative meta-analysis, as the primary goal of that analysis was to identify robust neurobiological differences, rather than to explore novel brain regions. A threshold of voxel-wise *p* < 0.0005 and cluster extent ≥ 20 voxels was used for meta-regression analyses given the exploratory nature (31). The between-study heterogeneity of the identified clusters was estimated with *I^2^* statistics, which represents the proportion of the total variation caused by study heterogeneity (32, 33). A value of 0% to 30% is interpreted as mild heterogeneity and a value > 50% indicates substantial heterogeneity, which should be treated with caution. To examine the reliability of the findings, we used jackknife sensitivity analyses which rerun the same meta-analysis iteratively with the removal of one study in one iteration (28). This analysis assesses the influence of individual studies on the pooled effect size. Moreover, we examined the possible publication bias by applying Egger’s test with funnel plots within SDM-PSI in which results with *p* < 0.05 indicated significant publication bias.

## 3 Results

### 3.1 Included studies and sample characteristics

In total, the systematic literature search identified 10 studies for NSSI (200 NSSI vs 309 controls) and 16 studies for SA (343 SA vs 630 controls) that met all inclusion criteria for the meta-analysis. The demographic and clinical information (e.g., sample sizes, age, sex, psychiatric condition etc.) and findings of individual studies are provided in **Supplementary Table 2** and **Supplementary Table 3** in **Supplementary Material**. The sample size-weighted *t-*test between the two groups revealed no significant difference in mean age (NSSI: 22.89 years, SA: 28.65 years; *t* = 1.29, *p* = 0.21) and female ratio (NSSI: 0.76, SA: 0.65; *t* = -0.96, *p* = 0.35).

### 3.2 Task-related fMRI abnormalities

#### 3.2.1 NSSI individuals versus controls

**Figure 2:**
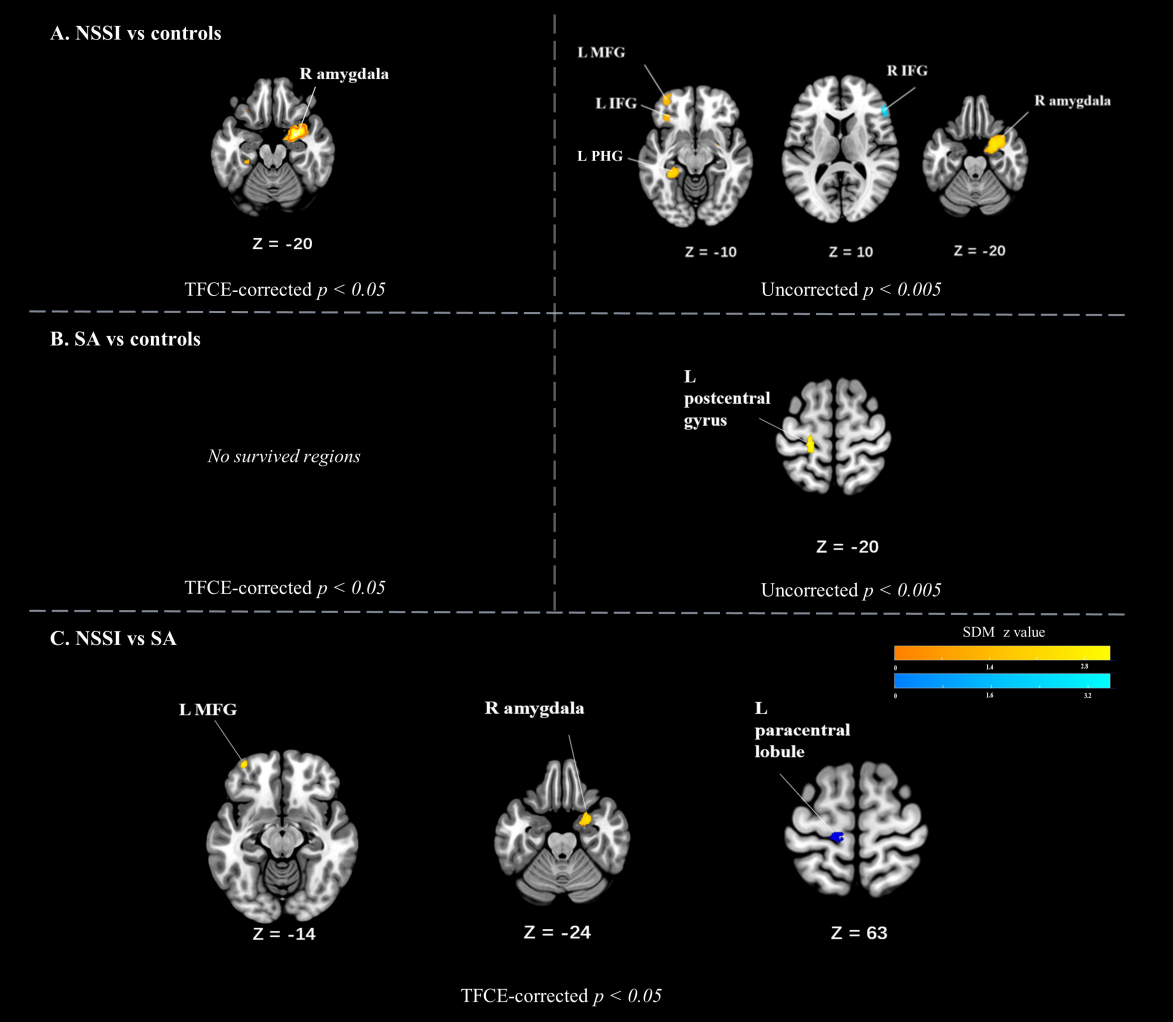
Brain activation alterations in NSSI and SA groups and their comparison. **(A)** Meta-analytic results for NSSI relative to controls. **(B)** Meta-analytic results for SA relative to controls. The left panel shows brain regions significant at TFCE-corrected *p* < 0.05, whereas the right panel shows brain regions significant at uncorrected *p* < 0.005 in separated meta-analyses. **(C)** Comparative meta-analytic results for NSSI (vs controls) in comparison to SA (vs controls) covarying for age and sex at threshold of TFCE-corrected *p* < 0.05. L, left; R, right; NSSI, nonsuicidal self-injury; MFG, middle frontal gyrus; SA, suicidal attempt. Warm color (orange) indicates increased activation. Cool color (blue) indicates decreased activation.

As shown in **Figure 2A** and **Table 1**, compared to controls, NSSI showed hyperactivation in the right amygdala (MNI coordinates: 26, 0, -20; peak *Z* value: 5.611; *k* = 463) at TFCE-corrected *p* < 0.05. No hypoactivation was found in NSSI individuals compared with controls. When applying a more liberal threshold (*p* < 0.005, uncorrected), NSSI individuals exhibited additional hyperactivation in left parahippocampal gyrus (PHG), MFG and IFG. Furthermore, hypoactivation was observed in the right IFG.

**Table 1:** Meta-analysis results for task-related fMRI studies in NSSI and SA.

MNI coordinates	SDM Z	Voxels	Regions	BA	Egger’s bias	Egger’s p
NSSI vs. Controls						
*TFCE-corrected p < 0.05*						
NSSI > Controls						
26,0,-20	5.611	463	Right amygdala	34	0.14	0.912
NSSI < Controls						
n.s.						
*Uncorrected p < 0.005*						
NSSI > Controls						
26,0,-20	5.611	835	Right amygdala	34	0.14	0.912
-30,-40,-8	4.494	252	Left parahippocampal gyrus	37	0.26	0.847
-38,58,-10	3.47	122	Left middle frontal gyrus, orbital part	46	1.37	0.332
-36,28,-14	3.323	97	Left inferior frontal gyrus, orbital part	47	0.48	0.763
NSSI < Controls						
54,28,10	-3.587	106	Right inferior frontal gyrus, triangular part	45	-1.63	0.217
SA vs. Controls						
*TFCE-corrected p < 0.05*						
SA > Controls						
n.s.						
SA < Controls						
n.s.						
*Uncorrected p < 0.005*						
SA > Controls						
-20,-32,64	3.275	61	Left postcentral gyrus	4	1.92	0.245
SA < Controls						
n.s.						
NSSI vs. SA (TFCE-corrected p < 0.05)						
NSSI > SA						
24,0,-24	2.501	128	Right amygdala	28	0.39	0.629
-36,56,-14	3.326	25	Left middle frontal gyrus, orbital part	47	0.5	0.542
NSSI < SA						
-16,-28,64	-2.147	37	Left paracentral lobule	4	0.39	0.640
Conjunction in NSSI and SA						
n.s.^a^						

^a^ No significant results at both TFCE-corrected *p* < 0.05 and uncorrected *p* < 0.005. BA, Brodmann’s area; MNI, Montreal Neurological Institute; n.s., not significant; NSSI, nonsuicidal self-injury; SA, suicide attempt; SDM, Seed-based d Mapping.

Bold values indicate the results of different group comparisons: NSSI vs. Controls, SA vs. Controls, NSSI vs. SA, and Conjunction in NSSI and SA.

#### 3.2.2 SA individuals versus controls

No significant differences were found between SA and controls at TFCE-corrected *p* < 0.05. At uncorrected *p* < 0.005, SA individuals showed regional-specific greater activation in the left postcentral gyrus (MNI coordinates: -20, -32, 64; peak *Z* value: 3.275; *k* = 61; **Table 1**; **Figure 2B**) than controls while no clusters of hypoactivation were found.

#### 3.2.3 Comparison of fMRI activation differences between NSSI and SA

After covarying for age and sex, significant activation differences were identified between NSSI and SA at TFCE-corrected *p* < 0.05 (**Table 1**; **Figure 2C**). Compared with SA individuals, NSSI-differentiating hyperactivation was found in the right amygdala (MNI coordinates: 24, 0, -24; peak *Z* value: 2.501; *k* = 128) and left MFG (MNI coordinates: -36, 56, -14; peak *Z* value: 3.326; *k* = 25). In contrast, SA group exhibited increased activation in left paracentral lobule (MNI coordinates: -16, -28, 64; peak *Z* value: -2.147; *k* = 37) relative to NSSI.

#### 3.2.4 Conjunction analysis between NSSI and SA

Conjunction analyses showed no overlapping activation between NSSI and SA groups at both TFCE-corrected *p* < 0.05 and uncorrected *p* < 0.005.

### 3.3 Sensitivity analyses

**Figure 3:**
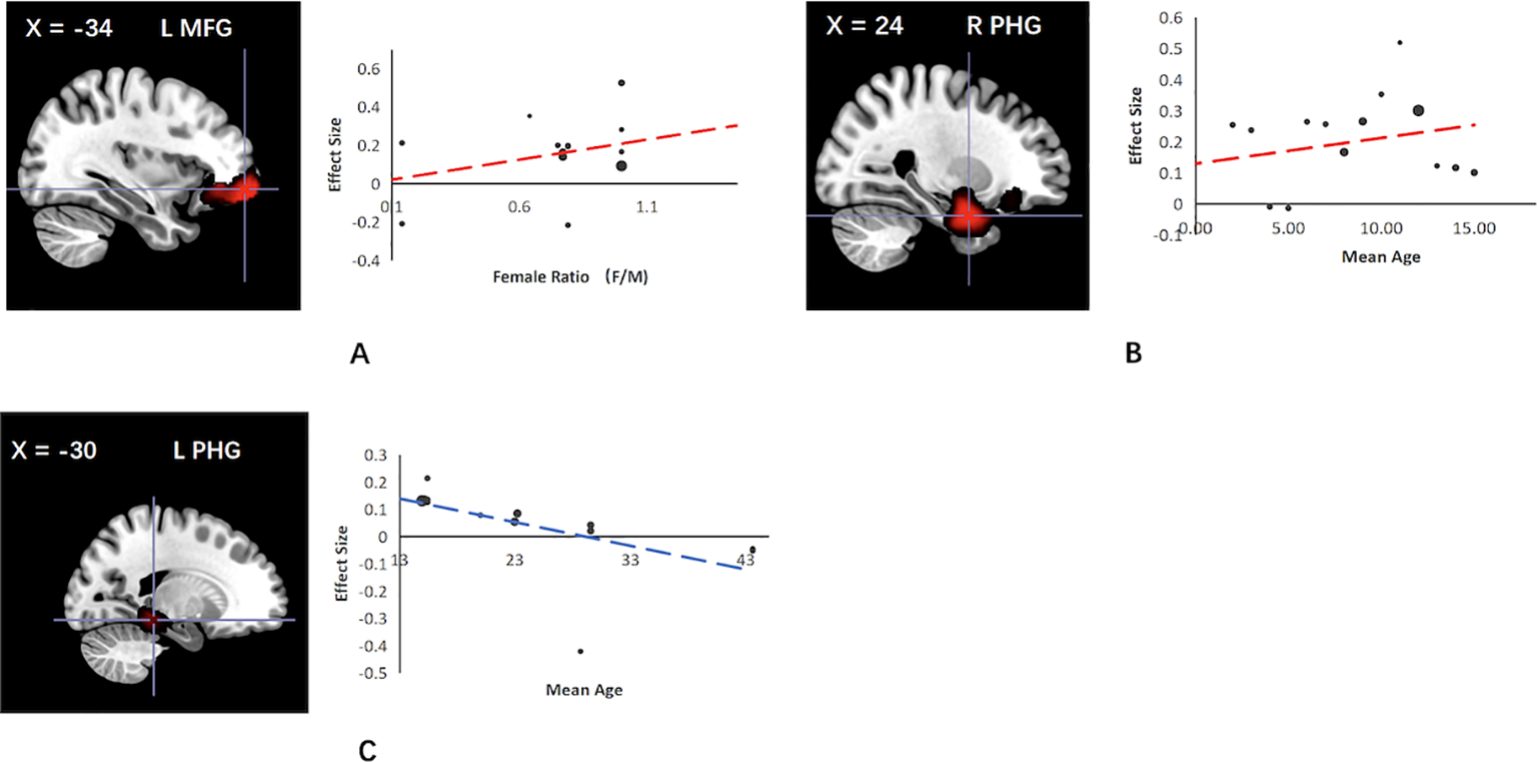
Results of meta-regression analyses. **(A)** Left MFG activation of NSSI is positively associated with female ratio. **(B)** Right PHG activation of NSSI is positively associated with age. **(C)** Left PHG activation of NSSI is negatively associated with age. F, female; M, male; MFG, middle frontal gyrus; NSSI, nonsuicidal self-injury; PHG, parahippocampal gyrus.

Within NSSI group, meta-regressions suggested that the higher proportion of female was associated with heightened activation in the left MFG (**Figure 3A**; MNI coordinates: -34, 56, -12; peak *Z* value: 4.132; *k* = 142). In addition, mean age of NSSI group was positively associated with activation in the right PHG (**Figure 3B**, MNI coordinates: 24, 0, -26; peak *Z* value: -3.324; *k* = 187) and negatively associated with activation in left PHG (**Figure 3C**, MNI coordinates: -30, -36, -12; peak *Z* value: -3.552; *k* = 167). No associations between activation alterations and confounding variables (age, sex and medication) were observed in SA.

Given potential neurobiological differences between adolescents and adults, we further conducted subgroup analyses separating pediatric and adult samples (see **Supplementary Table 4**). After TFCE correction, the pediatric NSSI subgroup showed increased activation in the right amygdala (MNI coordinates: 26, 2, -24; peak *Z* value: 5.315; *k* = 424) and left median cingulate (MNI coordinates: -28, 34, -12; peak *Z* value: 4.951; *k* = 21). In contrast, no significant activation alterations were observed in the adult NSSI subgroup. Neither the pediatric nor adult subgroup with SA demonstrated significant results. These results suggest that neurofunctional alterations associated with NSSI are primarily evident during developmental stages. Additional subgroup analyses showed no consistent or strong evidence for the influence of control type, psychiatric condition and task type (see **Supplementary Tables 5**-**S7**).

The results for heterogeneity statistics *I^2^* from the significant clusters indicated low to moderate heterogeneity (see **Supplementary Table 8**) across the main meta-analyses, suggesting that the findings are consistent and reliable across the included studies and are not influenced by inter-study methodological differences. The jackknife sensitivity analyses revealed that the main results observed in the meta-analyses are highly consistent (see **Supplementary Table 9**). Egger’s test did not detect significant funnel plot asymmetry, suggesting no evidence of publication bias (*p* > 0.05; **Table 1**; **Supplementary Figures 1**-**S6**).

## 4 Discussion

The current meta-analysis represents the first direct statistical comparison between NSSI an SA, providing robust evidence for a neurobiological divergence that distinguishes non-suicidal from suicidal SIB.NSSI individuals consistently and robustly exhibited increased activation in the right amygdala compared to both controls and those with SA, as well as heightened activation in the left MFG and reduced activation in the left paracentral lobule relative to SA group. SA showed less robust increased activation in the left postcentral gyrus relative to controls. No common functional abnormalities were found between NSSI and SA groups. These findings provide the first meta-analytic evidence demonstrating differential neurofunctional abnormalities in NSSI and SA, with NSSI characterized by excessive fronto-limbic engagement, and SA characterized by heightened somatosensory activation.

NSSI exhibited robust hyperactivation in the right amygdala. Amygdala hyperarousal has been frequently reported among NSSI subjects in previous studies (34–37). The amygdala is a region sensitive to relevant emotional stimuli (38), which aligns with the increased activation in amygdala observed in the subgroup meta-analysis of emotional task studies with a more liberal threshold. Previous studies have linked abnormal activation of amygdala in NSSI to greater vulnerability to psychiatric disorder, impulsivity and inappropriate emotion regulation attempts (36). Moreover, accumulating evidence suggests that amygdala-seed connectivity is altered in youth with NSSI, particularly with the ACC (22). Therefore, increased activation of amygdala in our study may indicate heightened emotional reactivity and reduced emotion regulation from the prefrontal cortex (34) in NSSI individuals. With a liberal threshold, we additionally identified hyperactivation in the left PHG, MFG and IFG, which aligns with the findings of a prior meta-analytic study on abnormal brain activity in NSSI using activation likelihood estimation approach (20), potentially suggesting a dysfunctional fronto-limbic circuit as a core neurobiological feature of NSSI (36). However, jackknife sensitivity analyses revealed that the left MFG and IFG were not consistently identified across all included studies for NSSI (**Supplementary Table 9**), indicating that these regional hyperactivations may be sensitive to the influence of specific large-sample studies. Consequently, these exploratory, uncorrected findings should be interpreted with caution and necessitate replication in future studies.

No significant functional alterations were observed in SA after correction. This result seems inconsistent with two previous meta-analytic findings (21, 39). However, although both found significant clusters, their results were obtained under a more lenient threshold without multiple comparison corrections. Moreover, these studies included both suicide attempters and ideators for analysis (21), or investigated suicide behaviors only in MDD patients (39). When we loosened the threshold, SA individuals showed increased activity in left postcentral gyrus, which is consistent with a recent meta-analysis showing that patients with suicidal ideation or suicide attempts exhibited increased activity in clusters located in parietal cortex (21). As part of the primary somatosensory cortex, the postcentral gyrus is engaged in somatosensory perception including processing sensations of touch, pressure, pain and proprioception (40, 41). The subgroup analysis further suggested that postcentral gyrus was more activated during emotional tasks in SA group compared with controls. Therefore, the heightened postcentral gyrus activation may be related to increased sensitivity to physical or emotional pain, as well as hyperreactivity to stress or negative stimuli in SA individuals. However, the results based on the exploratory threshold should be interpreted with caution and more rigorous design and analysis with large-scale datasets are required in future studies to establish more stable and replicable neuroimaging biomarkers in SA (42, 43).

The comparative meta-analysis revealed increased activation in right amygdala and left MFG as well as decreased activation in left paracentral lobule in NSSI relative to SA individuals. NSSI individuals often experience intense emotional responses, especially to negative or distressing stimuli (44). The hyperactivity in the amygdala may correspond to their heightened emotional reactivity, making it difficult to regulate strong emotions. In contrast, SA individuals may experience more emotional blunting or have diminished emotional reaction (45), which may link to decreased amygdala response relative NSSI. MFG has been involved in the cognitive regulation of emotion (46, 47). The reduced activation of the left MFG in SA relative to NSSI is in line with previous studies showing reduced dlPFC activation in response to value differences in individuals with suicide attempts (48, 49). MFG is a component of dlPFC, which plays a role in executive control, impulse control and decision-making (50, 51). Decreased MFG activation in SA might reflect an impaired ability to control and regulate thoughts related to impulsivity and emotion, leading to irrational decision-making and planning. By contrast, elevated activity in the MFG in NSSI suggests that individuals with NSSI may actively attempt to regulate their emotions, albeit through maladaptive strategies, which might reflect a compensatory effort to manage intense emotional distress (52). Our result parallels previous findings of elevated functional connectivity between the amygdala and paracentral lobule in SA individuals relative to controls (53). The paracentral lobule is a core part of the sensorimotor network, where increased internal activity has been linked to the integration and processing of somatic and visceral information, which are influenced by affective stimuli (54). Increased activation in the left paracentral lobule might reflect heightened integration of physical and emotional experiences in SA. These findings highlight distinct functional alterations underlying NSSI and SA, which may account for their differential abnormalities in emotion processing, regulation and embodiment.

The conjunction analysis did not yield overlapping brain alterations between NSSI and SA at both TFCE-corrected and uncorrected thresholds, which further supports that these behaviors are underpinned by divergent neural pathways. This finding challenged the assumption that NSSI and SA exist along a continuum of self-injurious behaviors (4, 55, 56). For example, according to the Gateway Theory (56), NSSI may serve as a precursor to more extreme behaviors (i.e., suicide attempts) by enhancing pain tolerance and reducing fear of death. However, not everyone with NSSI develop into SA, leading other researchers to argue for distinct psychological mechanisms such that NSSI can be differentiated from SA by more positive attitudes toward life, and more negative attitudes toward death (57). Therefore, the lack of convergence observed in our study may suggest neurobiological distinctions in self-injury related disorders, which may parallel behavioral differences between NSSI and SA. More original and meta-analytic evidence is needed to support this finding.

The meta-regression analysis revealed that female ratio was positively related to enhanced activation of left MFG in NSSI, which is consistent with a previous study of female NSSI individuals reporting abnormal activation in MFG (58). A meta-analysis found that the prevalence of NSSI among female adolescents is twice that of male adolescents (59). The modulatory effect of female ratio on MFG activation may reflect sex-specific patterns of cognitive control, rumination, or emotion dysregulation (60). The opposite direction of the correlational pattern between age and left and right PHG in NSSI might be attributed to their distinct patterns of functional connectivity with other brain regions. A previous study (61) found that the left PHG showed stronger functional connectivity with ACC, while the right PHG showed stronger connectivity with emotional processing regions including amygdala, midbrain and hippocampus. This lateralized connectivity profile may underlie differential age-related functional changes, with the right PHG potentially supporting compensatory mechanisms in emotional regulation, while the left PHG shows age-related decline in cognitive-affective integration. Our meta-regression results provided potential insights into the link between age and brain function in NSSI, which is further supported by the subgroup analyses separating pediatric and adult samples. Our subgroup analyses (**Supplementary Table 4**) revealed that the pediatric NSSI subgroup exhibited significant hyperactivity in the right amygdala and the left median cingulate, whereas these alterations were not significant in the adult cohort. This age-specific finding suggests that the neurofunctional abnormalities underlying NSSI, particularly in regions involved in emotional reactivity (amygdala) and the affective processing of pain (median cingulate, 62), may be more prominent during the transition from adolescence to young adulthood. The lack of significant results in the SA subgroups across ages further emphasizes the unique neurobiological trajectory of NSSI in early development, which is distinct from SA. These findings align with the previous perspective that the precursors and neurobiological correlates of NSSI and suicidality are heavily contingent upon developmental stage (63). Given that adolescent NSSI appears to follow a neurobiologically distinct trajectory from suicidal behavior, this finding has important implications for developmentally informed approaches to assessment and intervention, particularly for adolescents presenting with NSSI.

Several limitations should be considered in this meta-analysis. First, the limited number of NSSI studies constrains statistical power and may affect the robustness and stability of the identified clusters, necessitating a cautious interpretation. Future replications with larger, independent samples are necessary to confirm the generalizability and reliability of these neural markers. Second, the inclusion of varied paradigms (e.g., cognitive control, affective, and social tasks) may introduce potential variability. Although the low observed heterogeneity suggests a convergent neural signature, further research may benefit from domain-specific meta-analyses to isolate task-dependent neural patterns as more empirical studies become available. Third, this study was primarily based on peak coordinates rather than original maps, which may lower the sensitivity and power to detect robust neurofunctional alterations (64). Forth, the lack of robust SA findings and the adolescent−specific NSSI abnormalities limit claims of neural distinctness between NSSI and SA or generalizability to adults, warranting replication in larger and more age−diverse samples. Fifth, some clinical indicators, such as symptom severity and comorbidity are missing or inconsistent in original studies, which hampers us from investigating their effects on the results. Finally, the included NSSI/SA individuals had diverse diagnoses, and whether the results are influenced by other psychiatric conditions or unique to NSSI/SA behaviors remains further investigations. Given the limited number of each psychiatric condition, we only conducted a subgroup analysis for MDD. Future studies with larger sample size and well-powered design to examine the influence of important moderators such as psychiatric diagnosis and medication status will strengthen the findings and advance the field.

## 5 Conclusion

The current meta-analysis represents the first direct statistical comparison to investigate the common and differential neurofunctional dysregulations characterizing NSSI and SA. Our findings indicate that adolescent NSSI is associated with fronto-limbic activation abnormalities, whereas no significant findings emerged for adult NSSI or for SA subgroups under corrected thresholds, highlighting a unique neurobiological trajectory for NSSI during early development. Our results underscore the importance of age-informed risk stratification and targeted interventions for self-injurious behaviors, particularly in adolescents with NSSI.

## Data Availability

Publicly available datasets were analyzed in this study. This data can be found here: https://osf.io/39hkt/.
