# Supplementary material for: Comparative meta-analysis of task-related functional brain abnormalities in nonsuicidal self-injury and suicide attempt
Source: Front Psychiatry. 2026 Jul 7;17:1803658. doi: 10.3389/fpsyt.2026.1803658 (PMC13385046; doi:10.3389/fpsyt.2026.1803658)
Supplement: Supplementary file 1 [file DataSheet1.docx]

**Supplementary Material**

**Title**: Comparative meta-analysis of task-related functional brain abnormalities in nonsuicidal self-injury and suicide attempt

**Authors**: Lu Tang ^a,1^, Xiqin Liu ^a,b,c,1,*^, Yuanyuan Li ^a,c^, Nanfang Pan ^a,b^, Jianyu Li ^a,b^, Jian Zhou ^d^, Benjamin Becker ^e,f^, Qiyong Gong ^a,c,*^

**Affiliations:**

^a^ Department of Radiology, Huaxi Magnetic Resonance Research Center (HMRRC), Functional and Molecular Imaging Key Laboratory of Sichuan Province, West China Hospital of Sichuan University, Chengdu, Sichuan, 610041, China

^b^ Research Unit of Psychoradiology, Chinese Academy of Medical Sciences, Chengdu, Sichuan, 610041, China

^c^ Xiamen Key Laboratory of Psychoradiology and Neuromodulation, Department of Radiology, West China Xiamen Hospital of Sichuan University, Xiamen, Fujian, 361021, China

^d^ Department of Radiology, Affiliated Hospital of Guizhou Medical University, Guiyang, Guizhou, 550004, China

^e^ State Key Laboratory of Brain and Cognitive Sciences, The University of Hong Kong, Hong Kong, 999077, China

^f^ Department of Psychology, The University of Hong Kong, Hong Kong, 999077, China

^1^ The two authors contributed equally to this work.

***Corresponding Authors:**

Xiqin Liu, Ph.D., Huaxi Magnetic Resonance Research Center (HMRRC), Department of Radiology, Functional and Molecular Imaging Key Laboratory of Sichuan Province, West China Hospital of Sichuan University, 37 Guoxue Alley, Wuhou District, Chengdu, Sichuan, 610041, China

Qiyong Gong, M.D., Ph.D., Department of Radiology, West China Xiamen Hospital of Sichuan University, 699 Jinyuan Xi Road, Jimei District，Xiamen, Fujian, 361021, China

Fax: +86-28-85423503

Email: [qiyonggong@hmrrc.org.cn](mailto:qiyonggong@hmrrc.org.cn)

ORCID: <https://orcid.org/0000-0002-5912-4871>

**Table S1. Searching strategies in each database**

| **Database** |  | **Searching query** | **Number** |
| --- | --- | --- | --- |
| **Pubmed** | NSSI | (Self-Injurious Behavior[MeSH Terms] OR Behavior, Self-Injurious[Title/Abstract] OR Self Injurious Behavior[Title/Abstract] OR Self-Injurious Behaviors[Title/Abstract] OR Intentional Self Injury[Title/Abstract] OR Intentional Self Injuries[Title/Abstract] OR Self Injury, Intentional[Title/Abstract] OR Intentional Self Harm[Title/Abstract] OR Self Harm, Intentional[Title/Abstract] OR Nonsuicidal Self Injury[Title/Abstract] OR Nonsuicidal Self Injuries[Title/Abstract] OR Self Injury, Nonsuicidal[Title/Abstract] OR Deliberate Self-Harm[Title/Abstract] OR Deliberate Self Harm[Title/Abstract] OR Self-Harm, Deliberate[Title/Abstract] OR Self-Injury[Title/Abstract] OR Self Injury [Title/Abstract] OR Non-Suicidal Self Injury[Title/Abstract] OR Non Suicidal Self Injury [Title/Abstract] OR Non-Suicidal Self Injuries[Title/Abstract] OR Self Injury, Non-Suicidal [Title/Abstract] OR Self Harm[Title/Abstract] OR Harm, Self[Title/Abstract] OR Self-Destructive Behavior[Title/Abstract] OR Behavior, Self-Destructive[Title/Abstract] OR Self Destructive Behavior[Title/Abstract] OR Self-Destructive Behaviors[Title/Abstract]) AND (fMRI [Title/Abstract] OR functional magnetic resonance imaging[Title/Abstract] OR functional neuroimaging[Title/Abstract]) | 224 |
|  | SA | (Suicide[MeSH Terms] OR Suicides[Title/Abstract]) AND (fMRI[Title/Abstract] OR functional magnetic resonance imaging [Title/Abstract] OR functional neuroimaging[Title/Abstract]) | 167 |
| **web of science** | NSSI | TS= (Self-Injurious Behav* OR Self Injurious Behav* OR Intentional Self Injur* OR Intentional Self Harm OR Non$suicidal Self Injur* OR Deliberate Self-Harm OR Deliberate Self Harm OR Self-Injur* OR Self Injury OR Non-Suicidal Self Injur* OR Non Suicidal Self Injury OR Self Harm OR Self-Destructive Behav* OR Self Destructive Behav*) AND TS= (fMRI OR functional magnetic resonance imag* OR functional neuroimag*) | 1885 |
|  | SA | TS=(Suicid*) AND TS= (fMRI OR functional magnetic resonance imag* OR functional euroimag*) | 2465 |
| **Scopus** | NSSI | TITLE-ABS ((fMRI) OR (functional magnetic resonance imag*) OR (functional neuroimag*) ) AND TITLE-ABS ( ( self-injurious behav* ) OR ( self injurious behav* ) OR ( intentional self injur* ) OR ( intentional self harm ) OR ( non$suicidal self injur* ) OR ( deliberate self-harm ) OR ( deliberate self harm ) OR ( self-injur* ) OR ( self injury ) OR ( non-suicidal self injur* ) OR ( non suicidal self injury ) OR ( self harm ) OR ( self-destructive behav* ) OR ( self destructive behav* ) ) | 316 |
|  | SA | TITLE-ABS ((fMRI) OR (functional magnetic resonance imag*) OR (functional neuroimag*) ) AND TITLE-ABS ( ( suicid* ) ) | 470 |
| **EMBASE** | NSSI | (‘fMRI’ OR ‘functional magnetic resonance imag*’ OR ‘functional neuroimag*’) AND (‘self-injurious behav*’ OR ‘self injurious behav*’ OR ‘intentional self injur*’ OR ‘intentional self harm’ OR ‘non$suicidal self injur*’ OR ‘deliberate self-harm’ OR ‘deliberate self harm’ OR ‘self-injur*’ OR ‘self injury’ OR ‘non-suicidal self injur*’ OR ‘non suicidal self injury’ OR ‘self harm’ OR ‘self-destructive behav*’ OR ‘self destructive behav*’) | 196 |
|  | SA | (‘fMRI’ OR ‘functional magnetic resonance imag*’ OR ‘functional neuroimag*’) AND (‘Suicid*’ ) | 1212 |

Table S2. Sample characteristics and summary findings of task-related fMRI studies in NSSI included in the meta-analysis

| **Study** | **NSSI subjects** | | | | | **Control subjects** | | | | **Task (Type)** | | **Contrast** | | **Threshold** | | **Summary findings** | |  |
| --- | --- | --- | --- | --- | --- | --- | --- | --- | --- | --- | --- | --- | --- | --- | --- | --- | --- | --- |
|  | Psych.  cond. | Adult/  Pediat. | N (F) | Mean age (years) | Med. (%) | | PC/HC | N (F) | Mean age (years) | |  | |  | |  | |  | |
| Groschwitz et al. (2016) | MDD | Pediat. | 14 (11) | 15.4 | 21.4 | | PC/HC | 14 (11)/  15 (12) | 15.9/ 14.5 | | Cyberball task (emo.) | | Exclusion> inclusion | | uncorr *p* < 0.001, cluster extent ≥  10 | | NSSI > PC: R mPFC, L vlPFC, L PHG;  NSSI vs. HC: n.s. | |
| Lee et al. (2015) | SZ | Adult | 14 (2) | 43.6 | NA | | PC/HC | 14 (3)/  17 (3) | 38.9/ 37.9 | | Go/no-go task (cog.) | | No-go versus rest | | uncorr *p* < 0.001, cluster extent ≥ 5 | | NSSI > PC: R dlPFC, L vPCC; NSSI > HC: R dPCC;  NSSI < HC: R vlPFC, L vACC, R dlPFC, R thalamus; L vPCC | |
| Malejko et al. (2022) | MDD | Pediat. | 14 (11) | 15.4 | 71.4  (NSSI and PC) | | PC/HC | 13 (10)/  14 (11) | 16.2/ 14.4 | | Combined Go/No Go Eriksen flanker task (cog.) | | Incorrect Minus Correct In-congruent No Go Trials and Go trials | | uncorr voxel *p* ≤ 0.005, cluster *p* ≤ 0.05 | | NSSI > PC: M SMA;  NSSI < PC: M dACC, L/R IFG (pars triangularis and opercularis), L SMG, L IPC;  NSSI > HC: L precuneus;  NSSI < HC: R MFG, R IFG (pars triangularis and opercularis), R SMG, L IPC | |
| Moon et al. (2022) | NA | Adult | 28 (21) | 22.96 | drug-free | | HC | 38 (26) | 22.74 | | The REMT task (emo.) | | REMT > control | | uncorr *p* < 0.001, cluster extent ≥ 20 | | NSSI > HC: L SFG (medial part);  NSSI < HC: R angular gyrus | |
| Nam et al.  (2022) | NA | Adult | 26 (20) | 23.2 | drug-free  (> 1month) | | HC | 35 (25) | 22.7 | | Modified PRR task (cog.) | | Positive > neutral and negative > neutral | | uncorr *p* < 0.001, cluster extent ≥ 20 | | NSSI > HC: L/R IPL, R ITG, L calcarine, L insula, R thalamus; NSSI < HC: L/R IFG | |
| Osuch et al. (2014) | mood/anxiety disorder | Pediat. | 13 (10) | 20 | 61.5 | | PC | 15 (13) | 21 | | Pain stimuli (emo.) | | Cold > cool | | *p* < 0.005 (FDR) | | NSSI > PC: R midbrain, R pons, R PHG, R IFG, R STG, R amygdala, R culmen, R MFG; NSSI < PC: L/R cuneus, L precuneus | |
| Plener et al. (2012) | NA | Pediat. | 9 (9) | 15.2 | 11.1 | | HC | 9 (9) | 15 | | Picture-viewing task  (emo.) | | Baseline, arousal, self-injury picture content and valence | | *p* < 0.05 (FWE) | | NSSI > HC: L SPC, L/R hippocampus, L/R amygdala, L/R ACC, R cerebellum, L/R mOFC, L IPC, L IFG, L/R MOG;  NSSI < HC: L cuneus, L occipital cortex, R IFC | |
| Quevedo et al. (2016) | MDD | Pediat. | 50 (32) | 14.94 | 48 | | PC/HC | 36 (17)/  37 (18) | 14.77/ 14.49 | | Interpersonal self-processing task (cog.) | | All points of view, mother’s and classmates’ point of view | | cluster > 91.5 voxels, *p* < 0.05 (FWE) | | NSSI > PC + HC: L dlPFC, R precuneus, R mid-cingulate, R PCC, R SPL, L/R PHG, L/R fusiform, L/R hippocampus, R amygdala, R MTG | |
| Schmahl et al. (2006) | BPD | Adult | 12 (12) | 28.67 | drug-free  (> 4 weeks) | | HC | 12 (12) | 27.67 | | Heat and pain stimuli (emo.) | | Fixed temperature (early phase) and NRS 40 (early/late phase) | | *p* < 0.05 (FWE) | | NSSI > HC: L dlPFC;  NSSI < HC: R PPC, perigenual ACC, R amygdala, L temporal pole | |
| Vega et al. (2018) | BPD | Adult | 20 (20) | 29.55 | load index  2.5 ± 1.73 | | PC/HC | 20 (20)/  20 (20) | 31.2/  28.2 | | Gambling task (cog.) | | Gain > loss | | uncorr voxel *p* < 0.001, cluster *p* < 0.05 | | NSSI > PC: L OFC, L IFG (pars orbitalis), L MFG, R SFG; NSSI > HC: L OFC, L calcarine, R OFC, L/R ACG, L MTG | |

Abbreviations: ACG = anterior cingulate gyrus, BPD = borderline personality disorder, cog. = cognitive task, dACC = dorsal anterior cingulate cortex, dlPFC = dorsolateral prefrontal cortex, emo. = emotional task, F = female, FDR = false discovery rate correction, FWE = family-wise error correction, HC = healthy control, IFG = inferior frontal gyrus, IPC = inferior parietal cortex, IPL = inferior parietal lobe, ITG = inferior temporal gyrus, MDD = major depressive disorder, Med. = medication, MFG = middle frontal gyrus, mOFC = middle orbitofrontal cortex, MOG = middle occipital gyrus, mPFC = medial prefrontal cortex, MTG = middle temporal gyrus, N = sample size, NA = not available, n.s. = not significant, PC = patient control, Pediat. = pediatric (child/adolescent) sample, PHG = parahippocampal gyrus, PPC = posterior parietal cortex, Psych. cond. = psychiatric condition, RMET = Reading the Mind in the Eyes Test, SFG = Superior frontal gyrus, SMA = supplementary motor area, SMG = supramarginal gyrus, SPC = superior parietal cortex, SPL = superior parietal lobe, STG = superior temporal gyrus, SVC = small-volume correction, SZ = schizophrenia, uncorr = uncorrected, vACC = ventral anterior cingulate cortex, vlPFC = ventrolateral prefrontal cortex, vPCC = ventral posterior cingulate cortex.

Table S3. Sample characteristics and summary findings of task-related fMRI studies in SA included in the meta-analysis.

| **Study** | **SA subjects** | | | | | **Control subjects** | | | **Task (Type)** | **Contrast** | **Threshold** | **Summary findings** |
| --- | --- | --- | --- | --- | --- | --- | --- | --- | --- | --- | --- | --- |
|  | Psych.  cond. | Adult/  Pediat. | N (F) | Mean age (years) | Med. (%) | PC/HC | N (F) | Mean age (years) |  |  |  |  |
| Ai et al.  (2018) | MDD | Adult | 18 (14) | 37.72 | 77.8 | PC/HC | 54 (34)/  26 (13) | 37.61/  38.96 | Faces task and tower of London task (cog.) | NA | uncorr *p* < 0.001, *p* < 0.05 (FWE) | SA < PC: L/R FFG extending to lingual gyrus; SA > HC: L insula;  SA < HC: L/R FFG extending to lingual gyrus |
| Athanassiou et al. (2021) | SZ | Adult | 20 (2) | 18-60  (range) | 50 | PC/HC | 42 (6)/  22 (5) | NA | Emotional task (emo.) | Angry > control | uncorr *p* < 0.001, *p* < 0.05 (FDR) | SA > PC + HC: L MCG, L MFG, L precuneus, L hippocampus, L/R PMC, cerebellum, L PCA, L RO, L PMC |
| Aupperle et al. (2024) | MDD | Adult | 15 (13) | 32.36 | 0.87 | PC | 15 (14) | 31..13 | Episodic future thinking task (cog.) | Positive > negative | uncorr *p* < 0.005, *p* < 0.05 (Monte Carlo) | SA vs. PC: n.s. |
| Chase et al.  (2020) | MDD | Pediat. | 19 (14) | 15.63 | 94.7 | PC/HC | 22 (16)/  23 (14) | 16/  14.65 | Emotional faces task (emo.) | All emotional faces versus shapes | uncorr p< 0.001, *p* < 0.05 (FWE) | SA vs. PC: n.s;  SA < HC: R dlPFC, R SFG |
| Harms et al.  (2019) | MDD | Pediat. | 26 (21) | 14.57 | 100 | PC/HC | 39 (18) | 14.43 | Cyberball task (emo.) | Social vs non-social | *p* < 0.01 (FWE) | SA > HC: L/R ACC, L/R SFG, L/R MFG;  SA vs. PC + HC: n.s. |
| Jollant et al.  (2008) | MDD | Adult | 13 (0) | 40.3 | 30.8 | PC/HC | 14 (0)/  16 (0) | 43.9/  32.4 | Emotional faces response task (emo.) | Angry vs. neutral, mild angry vs. neutral, mild happy vs. neutral | corrected cluster *p* < 0.01 | SA > PC: R lOFC, R ACG, R cerebellum;  SA < PC: R SFG;  SA vs. HC: n.s. |
| Kim et al.  (2017) | NA | Adult | 14 (13) | 31.9 | NA | HC | 22 (13) | 33.6 | Emotional faces response task (emo.) | NA | uncorr *p* < 0.001, cluster extent ≥ 50 | SA > HC: L MFG, L IFG, L PCC, L thalamus |
| Miller et al.  (2023) | MDD | Pediat. | 34 (34) | 13.94 | 73.5 | PC/HC | 55 (55) | 12.12 | Emotion regulation task (emo.) | Before rejection decrease negative > look negative | corrected *p* <0.005 | SA > PC + HC: R frontal pole |
| Olie et al.  (2017) | MDD | Adult | 36 (36) | 39.48 | 33.3 | PC/HC | 41 (41)/  28 (28) | 37.6/  38.9 | Cyberball task (emo.) | ESE vs. INC | voxel *p* < 0.05 (FWE), cluster extent ≥ 10 | SA < PC + HC: L SMG, L pINS |
| Pan et al.  (2011) | MDD | Pediat. | 15 (11) | 16.2 | 66.7 | PC/HC | 15(8)/  14(6) | 15.87/  15.21 | Go/no-go task (cog.) | Go-no-go vs. Go | voxel *p* < 0.05, cluster *p* < 0.05 (FDR) | SA < PC: R ACG; SA vs. HC: n.s. |
| Pan et al. (2013a) | MDD | Pediat. | 15 (11) | 16.2 | 66.7 | PC/HC | 14 (7)/  13 (5) | 15.79/  15.15 | Iowa gambling task (cog.) | High risk vs. Low risk | uncorr *p* < 0.05 | SA < PC: L MTG, L hippocampus, R thalamus; SA > HC: L caudate |
| Pan et al. (2013b) | MDD | Pediat. | 14 (10) | 16.21 | 57.1 | PC/HC | 15 (8)/  15 (7) | 15.86/  15.27 | Facial emotion-processing task (emo.) | A50, AN, H100 and HN | voxel *p* < 0.05, cluster *p* < 0.05 (FDR) | SA > PC: R ACG, L/R PSC, L dlPFC, R MTG;  SA < PC: L FFG;  SA > HC: L PSC;  SA < HC: L PSC, R ACG, L MFG |
| Potvin et al.  (2018) | SZ | Adult | 13 (0) | 39.1 | 46.2 | PC/HC | 19 (0)/  21 (0) | 32.1/  32.1 | The balloon analog risk task (cog.) | The inflation event and the success event | voxel *p* < 0.001, cluster corrected *p* < 0.05 | SA > PC: R cerebellar declive, R lingual gyrus;  SA < PC: R MFG, R ACG; SA > HC: L STG;  SA < HC: R culmen, R cuneus, R MFG, R ACG, L MOG |
| Richard-Devantoy et al. (2016) | MDD | Adult | 26 (15) | 40.3 | drug-free  (before scanning) | PC/HC | 23 (15)/  28 (11) | 41.3/  33.8 | Go/no-go task (cog.) | Go vs. no-go and no-go vs. baseline | uncorr *p* < 0.001, cluster extent ≥ 10, *p* < 0.05 (FWE) | SA vs. PC: n.s.;  SA > HC: L/R IFG, R mPFC, L/R MFG, PCC, precuneus, L/R parietal regions near the superior marginal and angular gyrus; SA < HC: precuneus, PCC |
| Shaffer et al.  (2022) | BD | Adult | 19 (10) | 46.8 | 63.2 | PC | 20 (6) | 31 | Flashing checkerboard task (cog.) | Flashing checkerboard stimulus | *p* < 0.05 (FDR) | SA > PC: MOC, LOC, L cerebellar lobule V, L/R cerebellar lobule VI, R SOC, L STG |
| Silvers et al.  (2016) | BPD | Adult | 46 (46) | 28.98 | NA | PC | 14 (14) | 26.71 | Aversive personal memory recalling task (emo./cog.) | Distance +immerse > active baseline and distance > immerse | uncorr *p* < 0.005, *p* < 0.05 (FWE) | SA > PC: L lOFC;  SA < PC: L/R occipital gyri, R cuneus, R precuneus |

Abbreviations: ACC = anterior cingulate cortex, ACG = anterior cingulate gyrus, BD = bipolar disorder, BPD = borderline personality disorder, cog. = cognitive task, emo. = emotional task, F = female, FDR = false discovery rate correction, FFG = fusiform gyrus, FWE = family-wise error correction, HC = healthy control, IFG = inferior frontal gyrus, LOC = lateral occipital cortex, lOFC = lateral orbitofrontal cortex, MCG = median cingulate gyrus, MDD = major depressive disorder, Med. = medication, MFG = middle frontal gyrus, MOC = middle occipital cortex, MOG = middle occipital gyrus, MTG = middle temporal gyrus, N = sample size, NA = not available, n.s. = not significant, PC = patient control, PCA = precentral area, PCC = posterior cingulate cortex, Pediat. = pediatric (child/adolescent) sample, PFC = prefrontal cortex, pINS = posterior insula, PMC = primary motor cortex, PSC = primary sensory cortex, Psych. cond. = psychiatric condition, RO = rolandic operculum, STG = superior temporal gyrus, SZ = schizophrenia, SOC = superior occipital cortex, uncorr = uncorrected.

**Table S4. Subgroup-analysis results in the pediatric group and adult group**

| **MNI coordinates** | **SDM-Z** | **Voxels** | | **Regions** | | **BA** |
| --- | --- | --- | --- | --- | --- | --- |
| **TFCE-corrected *p* < 0.05** | | | | | | |
| **Pediat.** |  |  | |  | |  |
| NSSI > controls |  |  | |  | |  |
| 26,2,-24 | 5.315 | 424 | | Right amygdala | | 28 |
| -28,34,-12 | 4.951 | 21 | | Left median network, cingulum | | 37 |
| NSSI < controls |  |  | |  | |  |
| *n.s.* |  |  | |  | |  |
| *SA vs controls* |  |  | |  | |  |
| *n.s.* |  |  | |  | |  |
| **Adult** | | | | | |  |
| *NSSI vs controls* |  | |  | |  |  |
| *n.s.* |  | |  | |  |  |
| *SA vs controls* |  | |  | |  |  |
| *n.s.* |  | |  | |  |  |
| **Uncorrected *p* < 0.005** | | | | | |  |
| **Pediat.** | | | | | | |
| NSSI > controls |  |  | |  | |  |
| 26,2,-24 | 5.315 | 713 | | Right amygdala | | 28 |
| -28,34,-12 | 4.951 | 242 | | Left median network, cingulum | | 37 |
| NSSI < controls |  |  | |  | |  |
| 52,28,12 | -2.753 | 18 | | Right inferior frontal gyrus, triangular part | | 45 |
| SA > controls |  |  | |  | |  |
| -8,-28,62 | 3.141 | 141 | | Corpus callosum | | 4 |
| SA < controls |  |  | |  | |  |
| *n.s.* |  |  | |  | |  |
| **Adult** | | | | | |  |
| NSSI > controls |  |  | |  | |  |
| -38,54,-12 | 3.515 | 138 | | Left inferior frontal gyrus, orbital part | | 47 |
| NSSI < controls |  |  | |  | |  |
| *n.s.* |  |  | |  | |  |
| SA > controls |  |  | |  | |  |
| -8,-44,6 | 2.947 | 11 | | Left calcarine fissure/ surrounding cortex | | 29 |
| SA < controls |  |  | |  | |  |
| -28,-74,-12 | -3.849 | 170 | | Left fusiform gyrus | | 19 |
| 34,-68,-8 | -3.791 | 124 | | Right inferior network, inferior longitudinal fasciculus | | 19 |
| -36,-90,6 | -3.413 | 101 | | Left middle occipital gyrus | | 18 |
| 14,-78,30 | -3.368 | 50 | | Corpus callosum | | 18 |
| 26,54,0 | -3.163 | 11 | | Right middle frontal gyrus | | 11 |

Note: BA, Brodmann areas; MNI, Montreal Neurological Institute; n.s., no significant results; NSSI, nonsuicidal self-injury; Pediat., pediatric group; SA, suicide attempt; SDM, seed-based d mapping

**Table S5. Subgroup-analysis results in PCs and HCs**

| **MNI coordinates** | **SDM-Z** | **Voxels** | **Regions** | **BA** |
| --- | --- | --- | --- | --- |
| **PCs** | | | |  |
| *NSSI vs controls* |  |  |  |  |
| *n.s.^a^* |  |  |  |  |
| *SA vs controls* |  |  |  |  |
| *n.s. ^a^* |  |  |  |  |
| **HCs** | | |  |  |
| *NSSI vs controls* |  |  |  |  |
| *n.s. ^a^* |  |  |  |  |
| *SA vs controls* |  |  |  |  |
| SA > controls |  |  |  |  |
| -48,-4,0^b^ | 3.537 | 226 | Left superior temporal gyrus | 48 |
| SA < controls |  |  |  |  |
| *n.s. ^a^* |  |  |  |  |

Note: ^a^ no significant results at both TFCE corrected *p* < 0.05 and uncorrected *p* < 0.005; ^b^ results that thresholded at uncorrected *p* < 0.005, cluster size > 10 voxels. BA, Brodmann areas; HCs, healthy controls; MNI, Montreal Neurological Institute; n.s., not significant; NSSI, nonsuicidal self-injury; PCs, patient controls; SA, suicide attempt; SDM, seed-based d mapping

**Table S6. Subgroup-analysis results in MDD patients**

| **MNI coordinates** | **SDM-Z** | **Voxels** | **Regions** | **BA** |
| --- | --- | --- | --- | --- |
| NSSI > controls |  |  |  |  |
| -30,-36,-10^b^ | 4.762 | 207 | Left parahippocampal gyrus | 37 |
| NSSI < controls |  |  |  |  |
| *n.s.^a^* |  |  |  |  |
| SA > controls |  |  |  |  |
| *n.s. ^a^* |  |  |  |  |
| SA < controls |  |  |  |  |
| *n.s. ^a^* |  |  |  |  |

Note: ^a^ no significant results at both TFCE corrected *p* < 0.05 and uncorrected *p* < 0.005; ^b^ results that thresholded at uncorrected *p* < 0.005, cluster size > 10 voxels. BA, Brodmann areas; MDD, major depressive disorder; MNI, Montreal Neurological Institute; n.s., not significant; NSSI, nonsuicidal self-injury; SA, suicide attempt; SDM, seed-based d mapping

**Table S7. Subgroup-analysis results in emotional tasks and cognitive tasks**

| **MNI coordinates** | **SDM-Z** | **Voxels** | **Regions** | **BA** |
| --- | --- | --- | --- | --- |
| **Emotional tasks** |  |  |  |  |
| NSSI > controls |  |  |  |  |
| 24,2,-20^b^ | 3.511 | 143 | Right amygdala | 34 |
| -36,28,-14^b^ | 3.489 | 82 | Left inferior frontal gyrus, orbital part | 47 |
| NSSI < controls |  |  |  |  |
| *n.s.^a^* |  |  |  |  |
| SA > controls |  |  |  |  |
| -20,-32,62^b^ | 4.387 | 385 | Left postcentral gyrus | 3 |
| 4,50,4^b^ | 2.955 | 25 | Right superior frontal gyrus, medial | 10 |
| SA < controls |  |  |  |  |
| 12,40,50^b^ | -2.835 | 17 | Right superior frontal gyrus, dorsolateral | 9 |
| **Cognitive tasks** | | | |  |
| NSSI > controls |  |  |  |  |
| -38,52,-12^b^ | 2.923 | 33 | Left inferior frontal gyrus, orbital part | 47 |
| NSSI < controls |  |  |  |  |
| *n.s. ^a^* |  |  |  |  |
| SA > controls |  |  |  |  |
| -48,-4,0^b^ | 3.516 | 269 | Left superior temporal gyrus | 48 |
| SA < controls |  |  |  |  |
| -28,-74,-12^b^ | -3.899 | 175 | Left fusiform gyrus | 19 |
| 34,-70,-10^b^ | -3.916 | 138 | Right inferior network, inferior longitudinal fasciculus | 19 |
| -36,-92,4^b^ | -3.592 | 124 | Left middle occipital gyrus | 18 |
| 16,-76,30^b^ | -3.532 | 56 | Corpus callosum | 18 |
| 14,48,4^b^ | -3.22 | 29 | Right superior frontal gyrus, medial | 10 |
| 26,54,0^b^ | -3.559 | 23 | Right middle frontal gyrus | 11 |

Note: ^a^ no significant results at both TFCE corrected *p* < 0.05 and uncorrected *p* < 0.005; ^b^ results that thresholded at uncorrected *p* < 0.005, cluster size > 10 voxels. BA, Brodmann areas; MNI, Montreal Neurological Institute; n.s., not significant; NSSI, nonsuicidal self-injury; SA, suicide attempt; SDM, seed-based d mapping

**Table S8. Heterogeneity results (*I^2^*) in the main meta-analyses**

| **MNI coordinates** | **SDM Z** | **Voxels** | **Regions** | ***I^2^* (%)** |
| --- | --- | --- | --- | --- |
| NSSI vs. Controls | | | | |
| 26,0,-20^a^ | 5.611 | 463 | Right amygdala | 0.8 |
| -30,-40,-8^b^ | 4.494 | 252 | Left parahippocampal gyrus | 6.1 |
| -38,58,-10^b^ | 3.47 | 122 | Left middle frontal gyrus, orbital part | 13.5 |
| -36,28,-14^b^ | 3.323 | 97 | Left inferior frontal gyrus, orbital part | 8.0 |
| 54,28,10^b^ | -3.587 | 106 | Right inferior frontal gyrus, triangular part | 14.0 |
| SA vs. Controls | | | | |
| -20,-32,64^b^ | 3.275 | 61 | Left postcentral gyrus | 35.4 |
| NSSI vs. Controls | | | | |
| 24,0,-24^a^ | 2.501 | 128 | Right amygdala | 9.5 |
| -36,56,-14^a^ | 3.326 | 25 | Left middle frontal gyrus, orbital part | 0.8 |
| -16,-28,64^a^ | -2.147 | 37 | Left paracentral lobule | 11.2 |

Note: ^a^ results that survived TFCE correction (*p* < 0.05). ^b^ results that thresholded at uncorrected *p* < 0.005, NSSI = non-suicidal self injury, MNI = Montreal Neurological Institute, SA = suicide attempt, SDM = Seed-based d Mapping.

**Table S9. Jackknife sensitivity results of the studies included in the meta-analyses**

| **NSSI** | | | | | | |
| --- | --- | --- | --- | --- | --- | --- |
| **Removed study** | **R amygdala**^a^ | **L PHG** ^b^ | | **L MFG** ^b^ | **L IFG** ^b^ | **R IFG** ^b^ |
| Groschwitz et al. (2016) | YES | YES | NO | | YES | YES |
| Lee et al. (2015) | YES | YES | NO | | NO | YES |
| Malejko et al. (2022) | YES | YES | NO | | NO | YES |
| Moon et al. (2022) | NO | YES | NO | | NO | YES |
| Nam et al. (2022) | YES | YES | NO | | NO | YES |
| Osuch et al. (2014) | YES | YES | NO | | NO | YES |
| Plener et al. (2012) | YES | YES | NO | | NO | NO |
| Quevedo et al. (2016) | NO | NO | YES | | YES | YES |
| Schmahl et al. (2006) | YES | NO | NO | | NO | YES |
| Vega et al. (2018) | NO | YES | NO | | NO | YES |
| Total | 7/10 | 7/10 | 1/10 | | 2/10 | 9/10 |
| **SA** | | | | | | |
| **Removed studies** | **L postcentral gyrus** ^b^ | | | | | |
| Ai et al. (2018) | YES | | | | | |
| Athanassiou et al. (2021) | YES | | | | | |
| Aupperle et al. (2024) | YES | | | | | |
| Chase et al. (2020) | YES | | | | | |
| Harms et al. (2019) | YES | | | | | |
| Jollant et al. (2008) | YES | | | | | |
| Kim et al. (2017) | YES | | | | | |
| Miller et al. (2023) | YES | | | | | |
| Olie et al. (2017) | YES | | | | | |
| Pan et al. (2011) | YES | | | | | |
| Pan et al. (2013a) | YES | | | | | |
| Pan et al. (2013b) | YES | | | | | |
| Potvin et al. (2018) | YES | | | | | |
| Richard-Devantoy et al. (2016) | YES | | | | | |
| Shaffer et al. (2022) | YES | | | | | |
| Silvers et al. (2016) | YES | | | | | |
| Total | 16/16 | | | | | |

Note: ^a^ results that survived TFCE correction (*p* < 0.05). ^b^ results that thresholded at uncorrected *p* < 0.005. PHG = parahippocampal gyrus, MFG = middle frontal gyrus, IFG = inferior frontal gyrus.


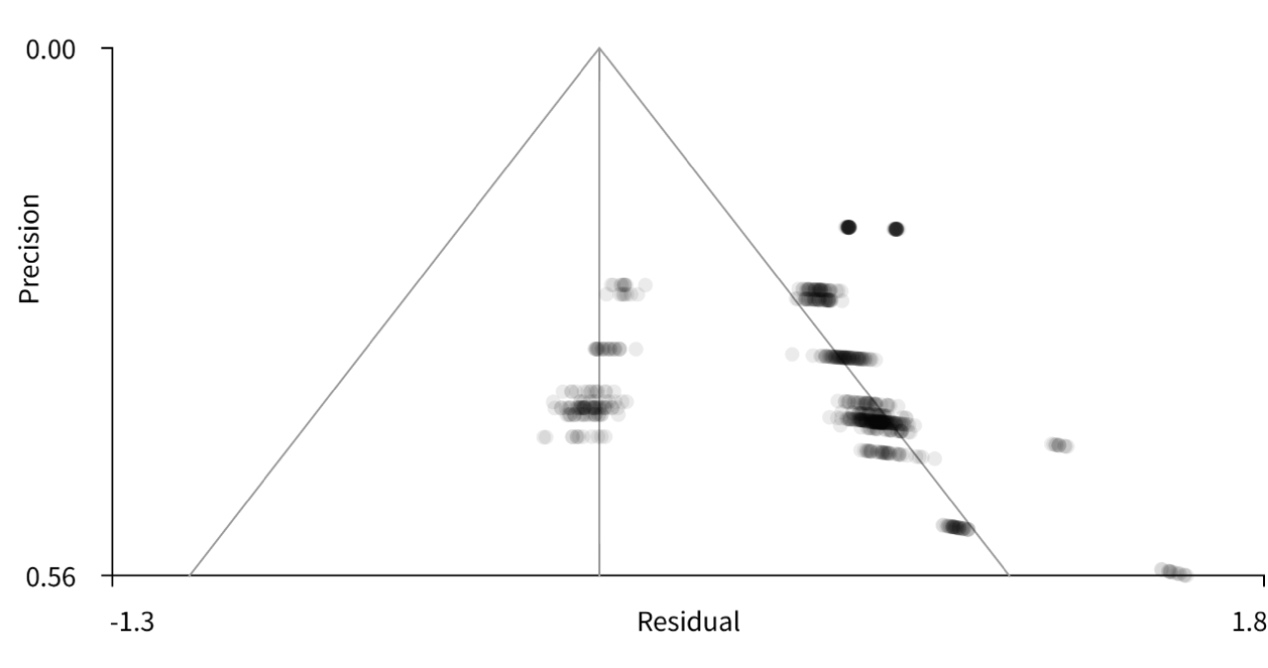


**Figure S1.** Funnel plots for the right amygdala (MNI: 26, 0, -20) in NSSI group at TFCE correction


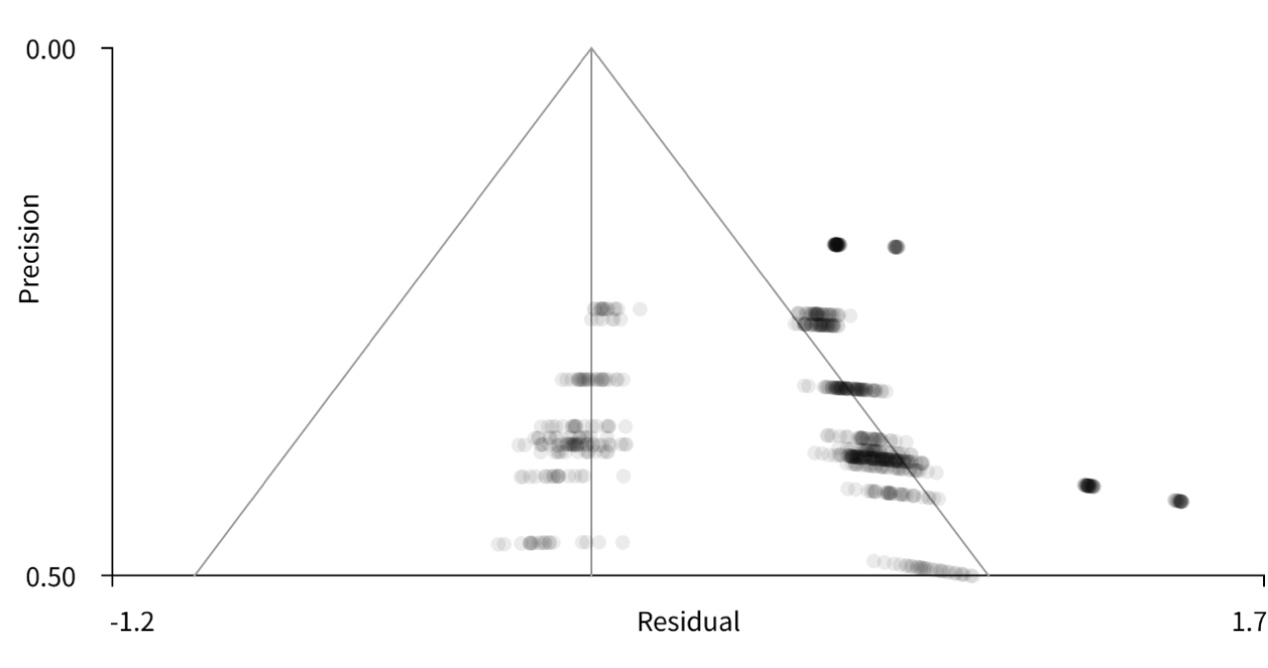


**Figure S2.** Funnel plots for the left PHG (MNI: -30, -40, -8) in NSSI group at uncorrected *p < 0.005*


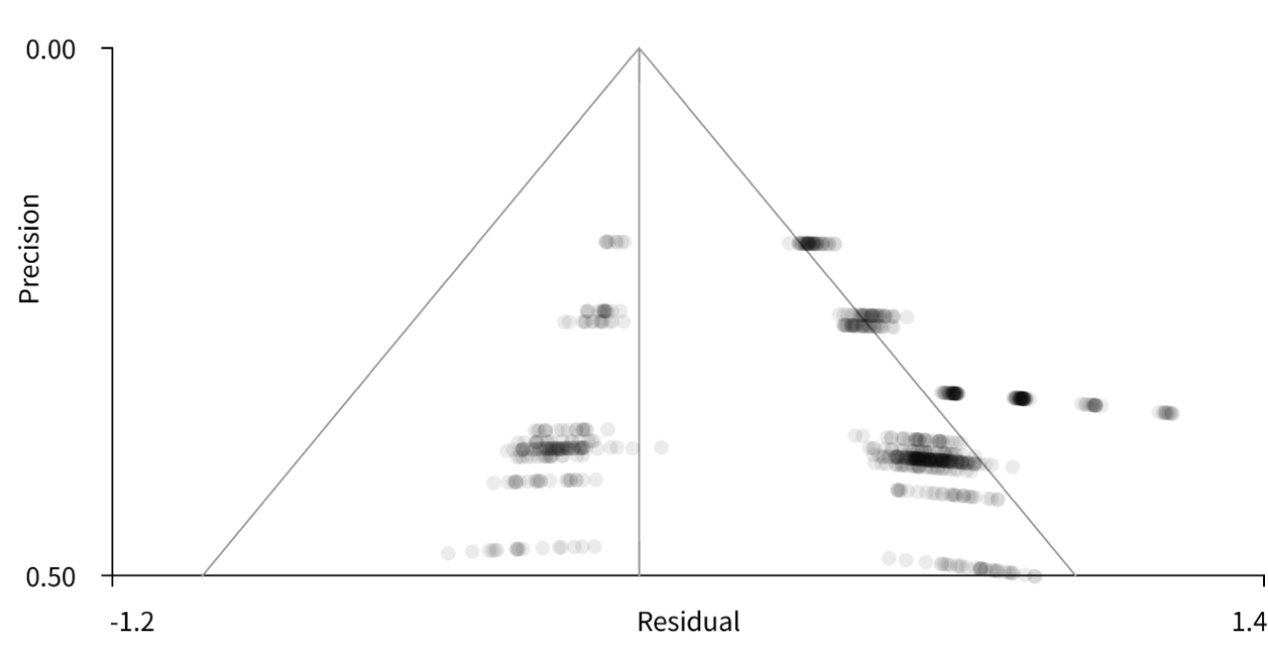


**Figure S3.** Funnel plots for the left MFG (MNI: -38, 58, -10) in NSSI group at uncorrected *p < 0.005*


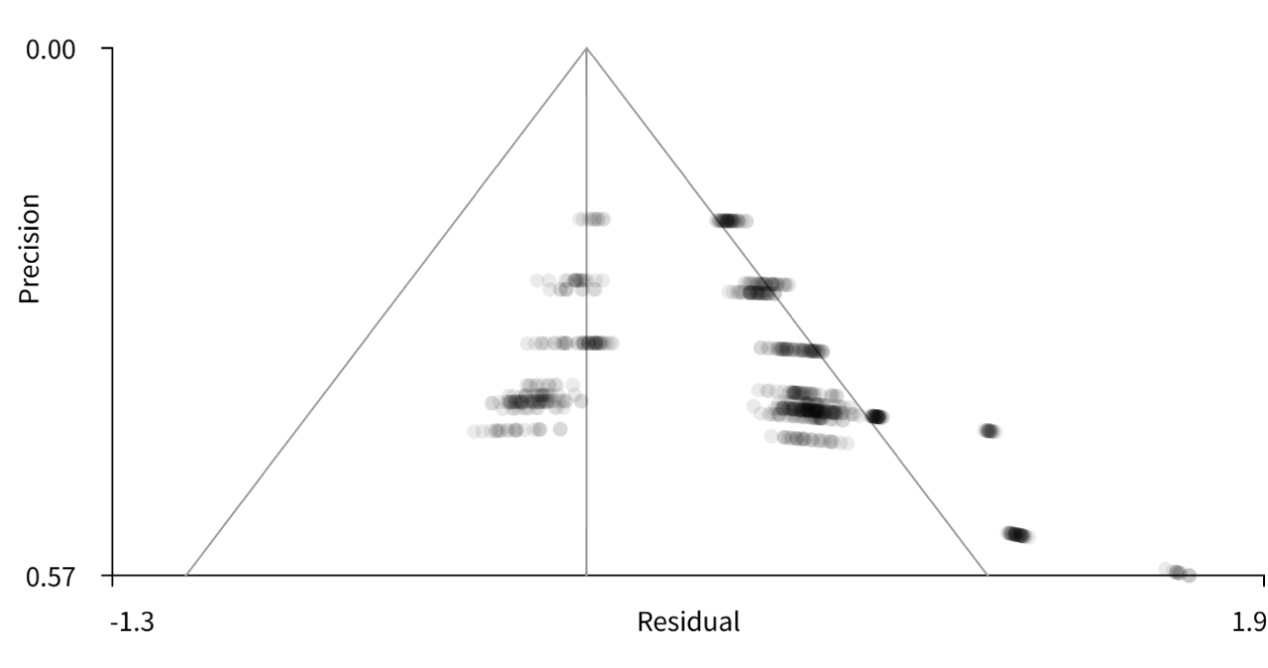


**Figure S4.** Funnel plots for the left IFG (MNI: -36, 28, -14) in NSSI group at uncorrected *p < 0.005*


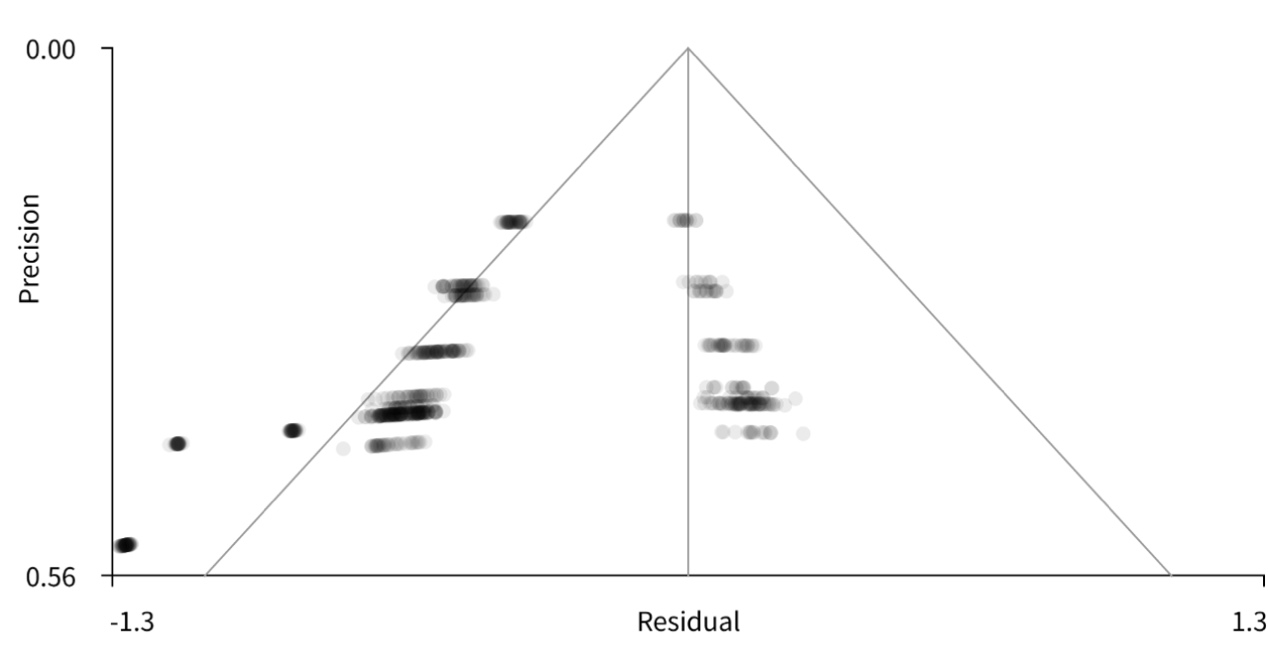


**Figure S5.** Funnel plots for the right IFG (MNI: 54, 28, 10) in NSSI group at uncorrected *p < 0.005*


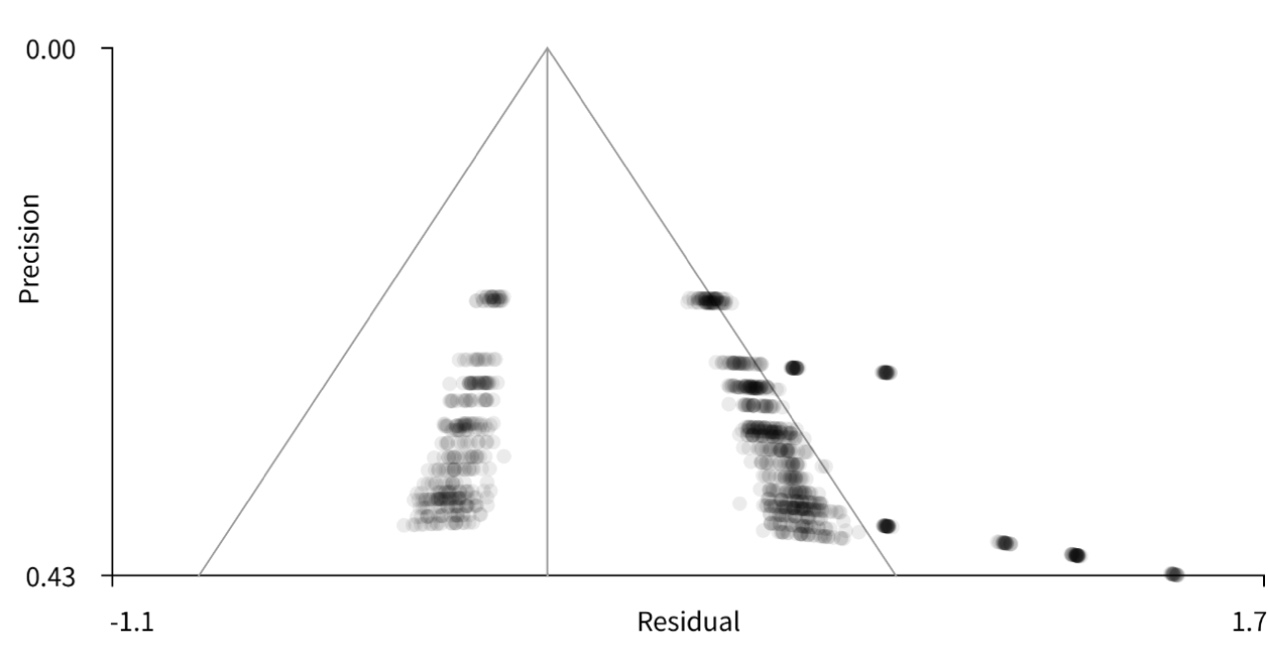


**Figure S6.** Funnel plots for the left postcentral gyrus (MNI: -20, -32, 64) in SA group at uncorrected *p < 0.005*
